# Insights into the Versatile Sulfur Metabolism of *Sulfurovum* sp. MH2-6 Isolated from Deep-Sea Hydrothermal Vent Environments

**DOI:** 10.3390/microorganisms14091916

**Published:** 2026-08-30

**Authors:** Liang Cui, Shasha Wang, Xuewen Gao, Rongfeng Hong, Zongze Shao, Lijing Jiang

**Affiliations:** 1 Key Laboratory of Marine Genetic Resources, Third Institute of Oceanography, Ministry of Natural Resources of China, Xiamen 361005, China; cuiliang@tio.org.cn (L.C.); gaogaoxw@stu.xmu.edu.cn (X.G.); 13808528741@163.com (R.H.); shaozz@163.com (Z.S.); 2 State Key Laboratory Breeding Base of Marine Genetic Resources, Third Institute of Oceanography, Ministry of Natural Resources of China, Xiamen 361005, China; 3 Fujian Ocean Innovation Center, Xiamen 361102, China

**Keywords:** sulfur disproportionation, *Sulfurovum*, hydrothermal vent, genomics, transcriptomics, chemolithoautotroph

## Abstract

In deep-sea hydrothermal ecosystems, inorganic sulfur compounds serve as key energy sources for microbes through oxidation, reduction, or disproportionation reactions. However, to date, the bacteria that disproportionate sulfur remain poorly understood. Here, we characterized the physiological and metabolic characteristics of *Sulfurovum* sp. MH2-6, which was isolated from hydrothermal sediments of the South Mid-Atlantic Ridge. Based on the results of 16S rRNA gene sequence, average nucleotide identity, and DNA–DNA hybridization value, strain MH2-6 belonged to the same species as *Sulfurovum mangrovi* ST1-3^T^. The isolate was able to grow chemolithoautotrophically using thiosulfate, sulfite, or sulfide as the sole energy source, and molecular oxygen as the sole electron acceptor. When using hydrogen as the sole energy source, this bacterium could utilize a wide range of electron acceptors, including oxygen, elemental sulfur, thiosulfate, nitrate, and sulfate. Various organic compounds also supported growth as carbon sources during hydrogen oxidation, suggesting a potential for chemolithomixotrophy. Notably, the isolate could grow via the disproportionation of thiosulfate and elemental sulfur in the presence of ferrihydrite. Further, genome analyses revealed that this bacterium contains a complete reductive citric acid cycle (rTCA) for carbon fixation, multiple hydrogenases, sulfur oxidation, reduction, and transfer enzymes, nitrogenase, and oxygen reductases. Transcriptomic comparisons between sulfur reduction and disproportionation conditions revealed that thiosulfate reductase, type IV sulfide: quinone oxidoreductase, and sulfite dehydrogenase were highly abundant in thiosulfate-disproportionating cultures, while rhodanese-like sulfurtransferases and sulfide dehydrogenase showed increased abundances when grown via elemental sulfur disproportionation. Together, these flexible energy- and carbon-utilizing strategies may enhance the persistence of MH2-6 in hydrothermal vent environments.

## 1. Introduction

Deep-sea hydrothermal vents are dark, high-pressure, and extreme ecosystems, where hot hydrothermal fluids mix with cold seawater, thereby generating sharp physical and chemical gradients. These gradients create a mosaic of ecological niches that provide diverse habitats that sustain microbial life [1,2]. As a typical dark ecosystem, chemolithoautotrophic microbes underpin primary production by converting geothermal chemical energy into biomass, thereby providing energy for higher trophic levels [3]. To achieve this, they harness energy from hydrogen, reduced sulfur compounds, carbon monoxide, methane, ammonia, nitrite, and various reduced minerals, driving the fixation of inorganic carbon into organic compounds and forming the foundation of vent food webs [4].

Sulfur species are widely distributed in hydrothermal systems and are central to microbial catabolism, primary production, organic matter turnover, and terminal degradation processes [5]. Various sulfur oxidation states (e.g., H_2_S, S^0^, SO_3_^2−^, S_2_O_3_^2−^) have been detected in hydrothermal chimneys, sediments, fluids, and the surrounding seawater, where they can serve as electron donors or terminal electron acceptors under aerobic and anaerobic conditions [6]. Microbial taxa involved in sulfide/sulfur oxidation, sulfur reduction and sulfate reduction have been well documented in hydrothermal habitats [7]. However, the isolates capable of sulfur disproportionation from these environments have only recently been described, despite thermodynamic conditions being favorable for this reaction [8]. In this process, intermediate inorganic sulfur compounds such as S^0^, S_2_O_3_^2−^, and SO_3_^2−^ are simultaneously reduced and oxidized into sulfide and sulfate [9]. To date, only six sulfur-disproportionating species have been isolated from hydrothermal vents, including thermophilic *Thermosulfuriphilus ammonigenes*, *Thermosulfurimonas dismutans*, and *Dissulfuribacter thermophilus*, mesophilic *Desulfolithobacter dissulfuricans* from deep-sea vents, and thermophilic *Thermosulfurimonas marina* and *Dissulfurirhabdus thermomarina* from shallow hydrothermal ecosystems [10,11]. Recently, five strains of *Sulfurimonas* and *Sulfurovum* from deep-sea hydrothermal vents have been verified to disproportionate thiosulfate and elemental sulfur [12], which expands the known taxonomic range of this process in vent sulfur cycling.

*Campylobacterota*, formerly assigned to *Epsilonproteobacteria*, represents key primary producers in deep-sea hydrothermal environments, comprising 66–98% of the microorganisms related to the substrates inhabiting certain vent fields [13]. Within this phylum, the genus *Sulfurovum* is widely distributed and often dominant across the global hydrothermal vents [14,15]. Notably, members of this genus are frequently found in hydrothermal plumes, as well as the endo- and/or epi-symbionts of hydrothermal animals [16]. These findings suggest that *Sulfurovum* taxa may be the key drivers of the biogeochemical cycles in hydrothermal ecosystems. To date, thirteen species belonging to this genus have been isolated from diverse marine habitats, including deep-sea hydrothermal vents and mangrove and coastal sediments [17]. To date, all characterized *Sulfurovum* isolates generally rely on reduced sulfur compounds or H_2_ as the sole electron donor, coupled with oxygen, nitrate, or elemental sulfur as the electron acceptors for chemolithoautotrophic growth [18]. However, the molecular machinery involved in sulfur metabolism in this genus remains poorly understood.

Here, we describe *Sulfurovum* sp. MH2-6, a bacterium isolated from hydrothermal sediment at the Xunmei hydrothermal field on the South Mid-Atlantic Ridge. Previous culture-independent analyses have revealed that members of the genus *Sulfurovum* are abundant in the hydrothermal habitats such as sediments and plumes of the South Mid-Atlantic Ridge [19,20]. We further report the diverse metabolic characteristics of this isolate including hydrogen oxidation, sulfur oxidation, reduction, and disproportionation, thereby gaining sufficient energy to synthesize biomass. Moreover, we integrated these physiological findings with transcriptomic data generated across four incubation conditions to identify candidate genes associated with sulfur disproportionation. This study provides new insights into the metabolic versatility of *Sulfurovum* in mediated-sulfur reactions under extreme environments.

## 2. Materials and Methods

### 2.1. Sample Collection, Enrichment, Purification, and Phylogenetic Analyses

The sediment samples were collected from the Xunmei hydrothermal field of South Mid-Atlantic Ridge (13°51′ W, 26°08′ S) with a ‘*Jiao-long*’ human-occupied vehicle at a depth of 2546 m at February 2024 during the COMRA DY83 cruise. Aboard the vessel, about 1 g of sediment samples were transferred into MMJ medium supplemented with 10 mM thiosulfate and 20 mM poorly crystalline Fe (III) [20], and then incubated at 32 °C under a gas phase of 80% N_2_/20% CO_2_ (200 kPa). Poorly crystalline Fe (III) was prepared by neutralizing FeCl_3_ with NaOH and subsequently desalting the precipitate with water [21]. MMJ medium contained the following constituents (per liter): 30 g NaCl, 0.14 g CaCl_2_·2H_2_O, 0.25 g NH_4_Cl, 4.18 g MgCl_2_·6H_2_O, 0.33 g KCl, 0.14 g K_2_HPO_4_, 12.5 mL of 1 M NaHCO_3_ solution, 1 mL of Wolfe’s vitamin mixture solution, and 10 mL trace element solution [22]. Following three rounds of sequential transfers and 10-fold serial dilutions in the same medium, a single morphological type was detected in the terminal growth-positive dilution (10^−8^). The purity of the culture was confirmed by microscopic examination and reconfirmed by 16S rRNA gene sequencing.

The genomic DNA was extracted according to a previously described method [23]. The 16S rRNA gene was amplified using primers Bac27F and 1492R [16], and sequence similarity was assessed with the EzTaxon-e server [24]. Closely related sequences were retrieved from GenBank for phylogenetic reconstruction. Multiple sequence alignment was performed with Clustalw v2.0.10, and the phylogenetic tree was constructed in MEGA v.6.0 using the maximum-likelihood algorithm [25].

### 2.2. Growth Characteristics

Cell morphology was examined with a light microscope (BX53, Olympus, Tokyo, Japan), and electron micrographs were observed with a transmission electron microscope (Tecnai G2 20, Thermo Scientific, Hillsboro, OR, USA). Growth with different conditions was monitored by direct cell counting using a phase contrast microscope (Eclipse 80i, Nikon, Tokyo, Japan).

All experiments described below were conducted in triplicate, and sterilized uninoculated medium served as a control. *Sulfurovum mangrovi* ST1-3^T^ was used as the reference strain in the cultivation experiments. The purification of the strain was confirmed again by 16S rRNA gene sequencing after the experiments. The growth temperature was examined at 4 °C, 10 °C, 15 °C, 20 °C, 25 °C, 28 °C, 30 °C, 32 °C, 35 °C, 37 °C, 45 °C, and 50 °C on MMJH medium. To assess pH tolerance, the medium was adjusted from pH 4.5 to 9.0 at 0.5-unit intervals using different buffers [26]. The salinity range was determined by varying the concentration of NaCl from 0% to 9% (wt/vol) at 0.5% intervals. The effect of oxygen on growth was assessed by setting different oxygen concentrations (0%, 1%, 2%, 4%, 6%, 8%, and 10% at 200 kPa and 20% at 100 kPa). When oxygen was omitted, 10 mM nitrate was supplied as a potential electron acceptor. Alternative electron donors were tested by replacing thiosulfate with sulfite (5 mM), tetrathionate (5 mM), thiocyanate (5 mM), sulfide (50–100 µM), or elemental sulfur (1% *w*/*v*) in MMJS medium under a gas phase of 72% N_2_/20% CO_2_/8% O_2_ (200 kPa). The molecular hydrogen was also examined in MMJH medium under 72% H_2_/20% CO_2_/8% O_2_ (200 kPa). For the electron acceptor, nitrate (10 mM), nitrite (5 mM), ferrihydrite (20 mM), ferric citrate (20 mM), elemental sulfur (1% *w*/*v*), and manganese oxide (200 µM) were tested in MMJH medium under 80% H_2_/20% CO_2_ (200 kPa).

Disproportionation of thiosulfate (10 mM), elemental sulfur (1% *w*/*v*), sulfite (5 mM), and tetrathionate (5 mM) was tested in sulfate-free MMJS medium under a gas phase of 80% N_2_/20% CO_2_ (200 kPa), in both the absence and presence of ferrihydrite (20 mM). Sulfur disproportionation was confirmed by the formation of sulfide and sulfate [27]. Sulfide production was monitored spectrophotometrically using the methylene blue method [28]. Thiosulfate and sulfate concentrations were determined by ion chromatography (DX-300, Dionex, Sunnyvale, CA, USA), with separation conducted on an IonPac AG19 column (4250 mm, Thermo Scientific, Sunnyvale, CA, USA) using 20 mM NaOH as the eluent at a flow rate of 1 mL/min. Fe (III) reduction was monitored by measuring Fe (II) using the ferrozine assay [29].

Heterotrophic growth was assessed in a NaHCO_3_-minus MMJH medium under 92% H_2_/8% O_2_ (200 kPa) using the following potential organic compounds as carbon source: 5 mM of formate, acetate, tartrate, citrate, propionate, pyruvate, and succinate, 5 mM each of 20 amino acids, 0.1% (*w*/*v*) peptone, tryptone, yeast extract, casein, starch, and casamino acids, 0.02% (*w*/*v*) galactose, fructose, maltose, glucose, sucrose, trehalose, and lactose. Further, these organic compounds were determined as a potential energy source replacing hydrogen in MMJ medium under a gas phase of 72% N_2_/20% CO_2_/8% O_2_ (200 kPa). Fermentative growth was also tested using the sulfur-free MMJ medium with these substrates under 80% N_2_/20% CO_2_ (200 kPa).

To confirm nitrogen fixation by the isolate, ^15^N_2_ tracer assays were performed in a 60 mL serum bottle containing 10 mL of the nitrogen-free MMJH medium under 72% H_2_/20% CO_2_/8% O_2_ (200 kPa) [30]. Immediately before inoculation, 10 mL of ^15^N_2_ (99.9 atom%, Germany) was added to the medium. Control assays were conducted by using ^15^N_2_-free medium with or without inoculation. The cells were collected during the late exponential growth phase by centrifugation (10,000 rpm, 4 °C, 20 min), and then washed twice with 20 mM Tris buffer prepared in artificial seawater, and freeze-dried overnight [31]. Nitrogen isotope ratios were analyzed by isotope ratio mass spectrometer couple with an elemental analyzer (Flash EA 2000, Thermo Scientific, Waltham, MA, USA).

### 2.3. Genome Sequencing and Analyses

The whole-genome sequencing was performed by Tianjin Biochip Corporation (Tianjin, China) on the Pacific Biosciences (PacBio) sequencing platform using the single-molecule real-time (SMRT) technology. After reads filtering, high-quality paired-end reads were assembled into a circular genome with SOAPdenovo v.2.04 [32]. The G+C content was calculated from the genome sequence. Gene annotation was performed by Rapid Annotation using Subsystem Technology (RAST) [33], and the automated Prokaryotic Genomic Annotation Pipeline (PGAP). The metabolic pathway was analyzed by searching against the Kyoto Encyclopedia of Genes and Genomes (KEGG) database by KEGG Automatic Annotation Server (KAAS). The average nucleotide identity (ANI) and in silico DNA–DNA hybridization (dDDH) between genomes were determined using the EzTaxon-e server [24] and the Genome-to-Genome Distance Calculator [26], respectively. Comparative genome analysis was determined using the BPGA v.1.3 [34] pipeline, based on orthologous groups of proteins with sequence similarity (*E*-value < 10^−5^, >50% coverage).

### 2.4. Transcriptomic Analysis

Cells of the isolate cultivated under four different growth conditions of thiosulfate and elemental sulfur reduction with hydrogen as the energy source, as well as thiosulfate and elemental sulfur disproportionation, were harvested during the late exponential growth phase, and centrifuged at 12,000 rpm at 4 °C for 10 min. The resulting pellets were immediately frozen in liquid nitrogen. Total RNA was extracted with an RNA extraction kit (Qiagen, Hilden, Germany) according to the manufacturer’s instructions [35]. RNA quality and concentration were determined by gel electrophoresis and a NanoDrop1000 spectrometer (Thermo Scientific, Wilmington, DE, USA). The transcriptome sequencing was performed by an Illumina Hiseq sequencing platform at Novogene Co., Ltd in Beijing, China. Raw reads were quality-filtered to remove adapter-contaminated reads, poly-N-containing sequences, and low-quality reads. The resulting clean reads were subsequently mapped to the isolate genome using Bowtie v0.12.8 [36]. Gene expression was normalized as fragments per kilobase of coding sequence per million reads (FPKM).

### 2.5. Statistical Analysis

The significant differences among groups were subjected to one-way analysis of variance (one-way ANOVA) and multiple comparisons using the SPSS 19.0 program. A statistical significance was defined in our study by *p* < 0.05 (indicated by * in all figures) or *p* < 0.01 (indicated by ** in all figures).

## 3. Results and Discussion

### 3.1. Isolation, Morphology, and Phylogeny

**Figure 1:**
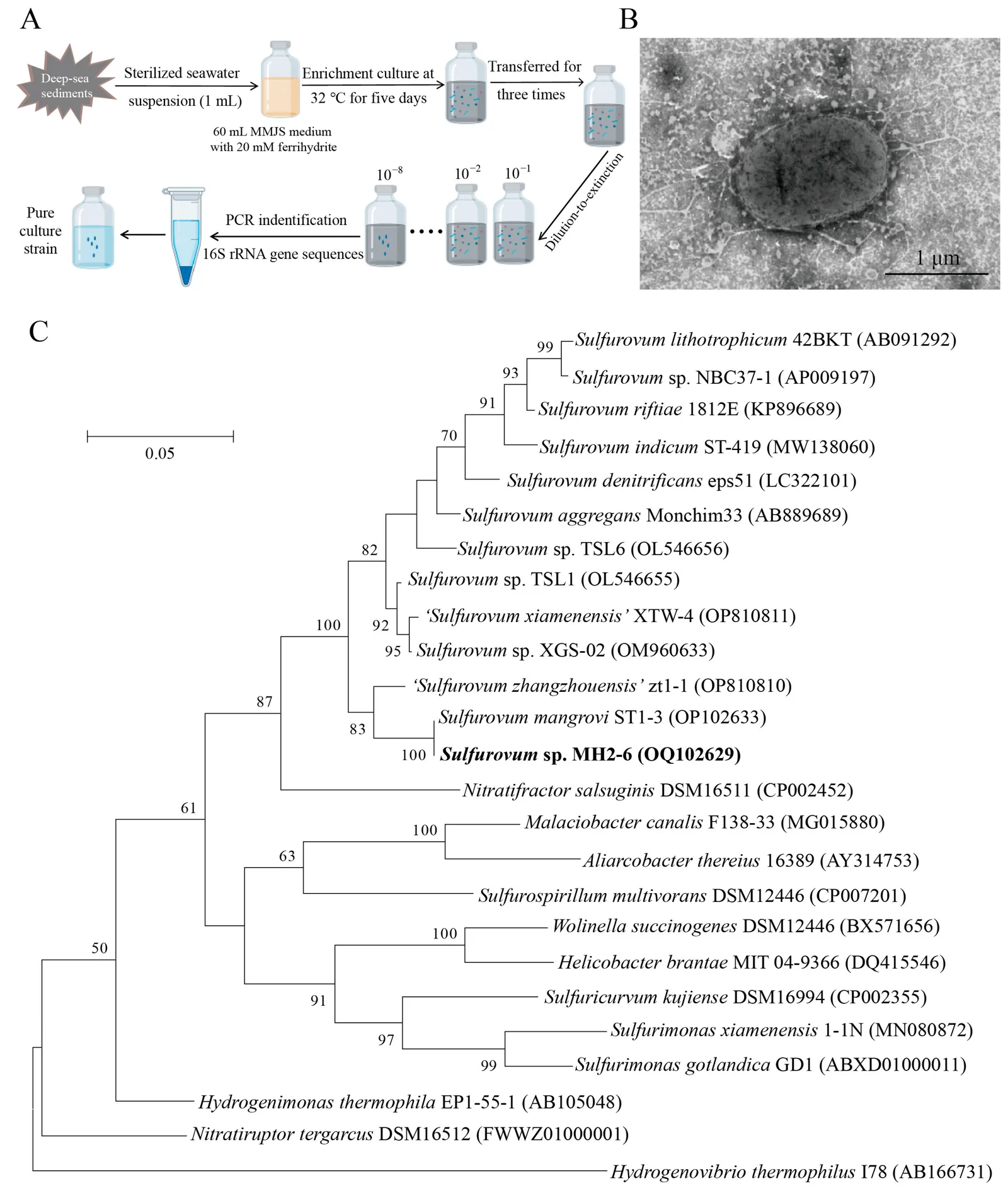
Isolation, morphology, and phylogenetic analyses of *Sulfurovum* sp. MH2-6. (**A**) Diagram showing the strategy used to isolate deep-sea sulfur-disproportionating bacteria. (**B**) Transmission electron microscopy image of a cell of strain MH2-6. Bar, 1.0 µm. (**C**) Phylogenetic placement of strain MH2-6 within the genus *Sulfurovum*, based on 16S rRNA gene sequences. Bootstrap values are based on 1000 replicates. Bar, 0.02 substitutions per nucleotide position.

Enrichment cultures were grown in liquid MMJS medium with CO_2_/HCO_3_^−^ as the carbon source, thiosulfate as the sole substrate, and ferrihydrite as a sulfide scavenger (Figure 1A). After 5 days of incubation, the production of H_2_S was indicated by a color change, wherein ferrihydrite reacted with H_2_S, yielding black ferrous sulfide precipitates. Microscopic observation revealed the presence of different morphological types of microorganisms, including coccoid, rod-shaped, curved, and spiral cells. Subsequent purification was achieved through three successive rounds of transfers and the dilution-to-extinction technique at 32 °C. The resulting isolate was consequently designated as strain MH2-6. The cells of strain MH2-6 were Gram-negative, non-motile, rod-shaped, around 0.2–0.6 µm in width and 0.7–1.5 µm in length (Figure 1B). Spore or flagellum was not observed microscopically in this study, which is consistent with the previous report of the genus *Sulfurovum* [16]. A blast search of the 16S rRNA gene sequences showed that the isolate was closely related to *S*. *mangrovi* ST1-3^T^ with 99.92% sequence similarity (Figure 1C), suggesting that the two strains belong to the same species within the genus *Sulfurovum*.

### 3.2. Physiological Characteristics

Growth assays demonstrated that the isolate grew over a range of temperatures (4–50 °C), salinities (1–5% NaCl), pH (5.0–8.0), and oxygen concentrations (0–20%). Optimum growth occurred at 32 °C, 3% NaCl, pH 6.0, and 8% O_2_ (Table 1). Moreover, this strain also exhibited good growth under atmospheric concentrations in the headspace. Under the optimum growth conditions with hydrogen as the energy source, the maximum cell yield was approximately 8–9 × 10^8^ cell mL^−1^ and the doubling time was 2.5 h (Table 1).

**Figure 2:**
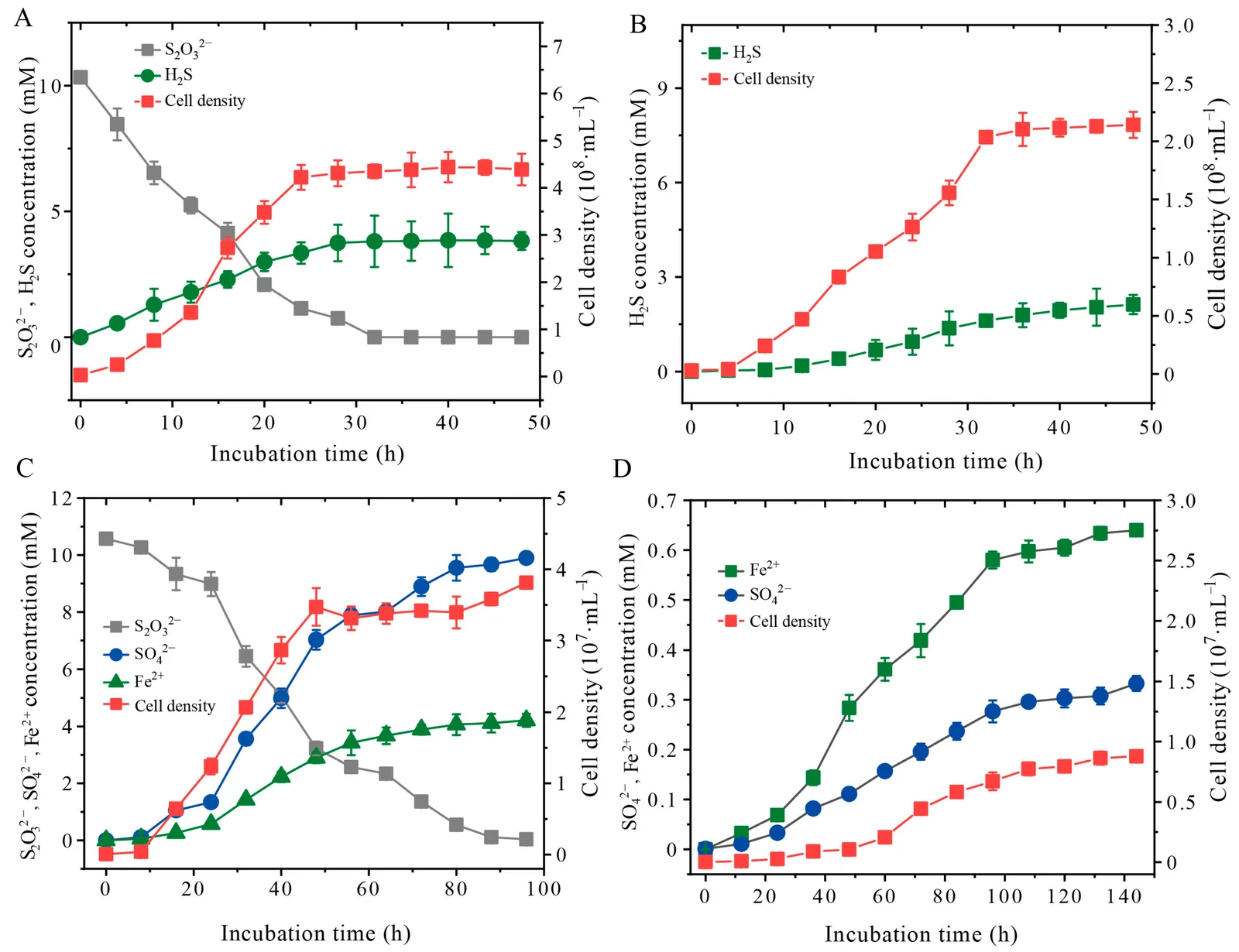
The growth characterization of *Sulfurovum* sp. MH2-6 with hydrogen as the electron donor and thiosulfate (**A**) or elemental sulfur (**B**) as the sole electron acceptor, and thiosulfate (**C**) and elemental sulfur (**D**) disproportionation in the presence of ferrihydrite, respectively. Error bars indicate the standard deviation from triplicates.

The chemoautotrophic growth tests revealed that strain MH2-6 could utilize H_2_ as an electron donor, coupled with a variety of compounds as terminal electron acceptors, including oxygen, elemental sulfur, thiosulfate, nitrate, and sulfate (Table 1). Among them, strain MH2-6 had the highest cell density when using oxygen as the sole electron acceptor, followed by thiosulfate (4.39 × 10^8^ cells mL^−1^) and elemental sulfur (0.61 × 10^8^ cells mL^−1^) (Figure 2A,B). After two days of incubation under the latter two conditions, strain MH2-6 could respectively produce 3.85 and 2.15 mM sulfide. Growth was also observed when nitrate and sulfate served as the electron acceptor with hydrogen as the electron donor, accompanied by the production of nitrite and sulfide, respectively (Figure S1 and Figure S2). In addition, strain MH2-6 was able to grow with thiosulfate, sulfide, and sulfite as the energy source, coupling oxygen as the sole electron acceptor. However, biomass yields were much lower under these conditions (1.1–1.3 × 10^7^ cells mL^−1^) than when hydrogen served as the sole energy source. These results indicate that hydrogen appears to be the preferred energy substrate for this bacterium.

**Figure 3:**
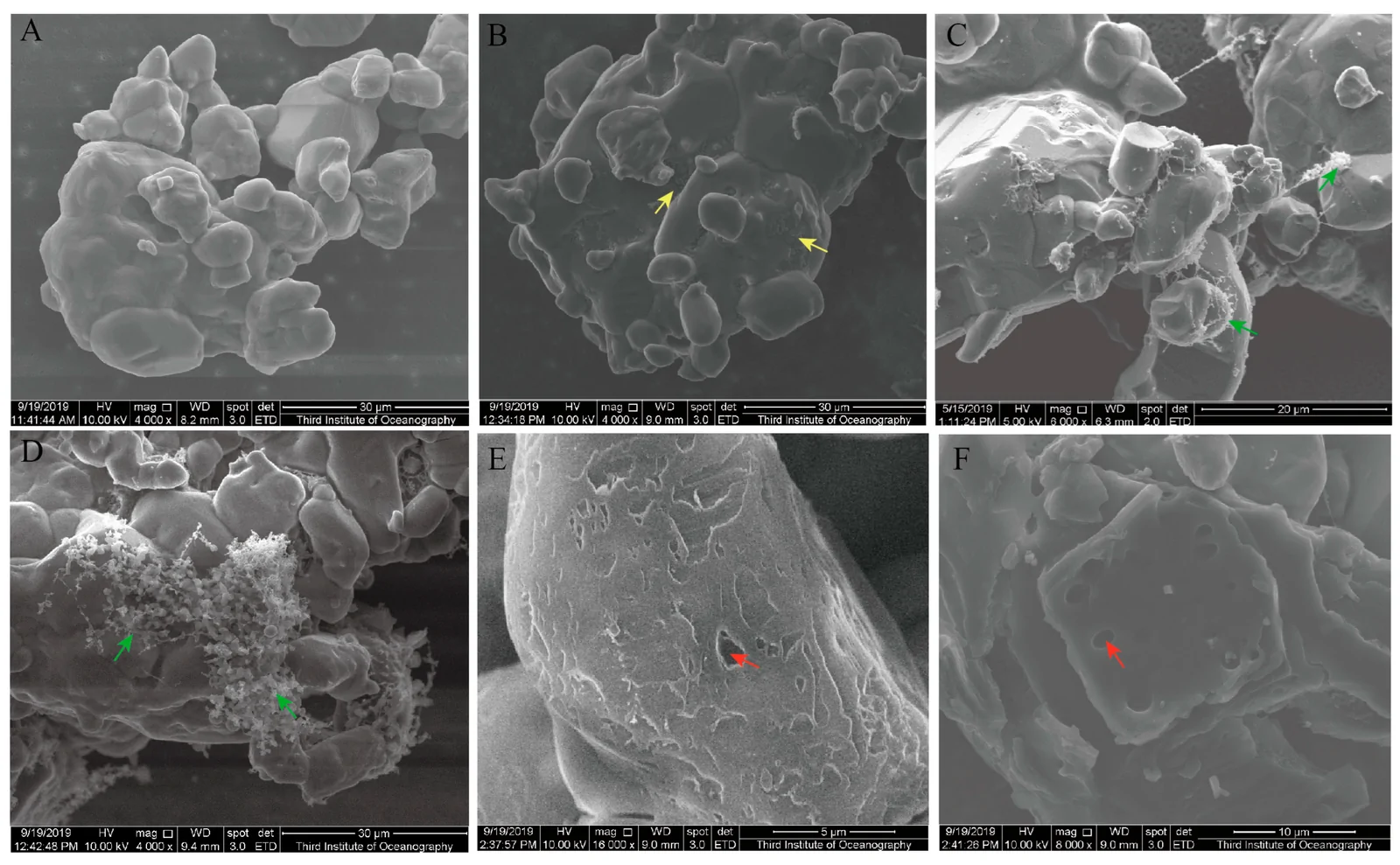
Scanning electron micrographs of sulfur granules from batch cultures of *Sulfurovum* sp. MH2-6 during elemental sulfur disproportionation. (**A**) Smooth sulfur granules in uninoculated medium; (**B**–**E**) Sulfur granules after 24 h of incubation; and (**F**) Sulfur granules after one week of incubation. Yellow arrows indicate bacterial cells, green arrows indicate the filamentous viscous substances, and red arrows indicate the dents on the surface of bulk sulfur granules.

Strain MH2-6 could grow via the disproportionation of thiosulfate and elemental sulfur but not sulfite, in the presence of ferrihydrite (Figure 2C,D). Approximately 10.57 mM thiosulfate was disproportionated to 9.89 mM sulfate and 4.21 mM Fe (II) over six days of incubation (Figure 2C). The nearly 1:1 ratio of the sulfate formed to the thiosulfate consumed was in accordance with the theoretical stoichiometry of the reaction: S_2_O_3_^2−^ + H_2_O = SO_4_^2−^ + HS^−^ + H^+^ [20]. Sulfide was sequestered as Fe-sulfide precipitates, whereas no dissolved sulfide could be detected. The maximal cell density was 3.81 × 10^7^ cells mL^−1^, and the doubling time was 1.7 days (Figure 2C). For elemental sulfur, the cell numbers reached a maximum of 0.88 × 10^7^ cells mL^−1^ over a six-day incubation (Figure 2D). Although elemental sulfur could not be quantified, the growth was coupled with the production of approximately 0.64 mM Fe (II) and 0.33 mM sulfate, yielding a ratio of 1.9–2.0:1, which is consistent with the stoichiometry of the reaction: 3S^0^ + 2Fe(OH)_3_ = SO_4_^2−^ + 2FeS + 2H^+^ + 2H_2_O [37]. During the process, a black non-magnetic precipitate was also present. As the controls, no such change in the sulfate and sulfide concentrations was observed. Furthermore, scanning electron microscopy revealed that a large number of cells were attached to the surface of the sulfur particles (Figure 3B), and much more filamentous viscous substances, which might be components of biofilms, were also observed (Figure 3C,D). Moreover, relative to the uninoculated control (Figure 3A), obvious dents were observed on the sulfur particle surface (Figure 3D). As the culture time increased, larger and deeper pits were ubiquitous on the surface of elemental sulfur (Figure 3E). These results indicate that direct physical contact and biofilm formation can facilitate the degradation of elemental sulfur in *Sulfurovum* species.

Heterotrophic growth assays indicated that strain MH2-6 could not utilize any of the tested organic carbons as the sole energy source. However, when hydrogen was supplied as the energy source, it could grow with various organic compounds as the carbon source, including tryptone, yeast extract, casein, citrate, glucose, tartrate, propionate, succinate, and fructose (Table 1). Among them, high biomasses (3.0–5.0 × 10^8^ cells mL^−1^) were achieved for cells with yeast extract, tryptone, succinate, glucose, or fructose as a carbon source (Figure S3), but the biomasses were lower than those observed under chemoautotrophic conditions. Other organic compounds, including amino acids, peptone, starch, pyruvate, formate, acetate, lactose, sucrose, galactose, maltose, and trehalose, could not support the bacterial growth. These results suggest that strain MH2-6 tends to prefer a chemolithoautotrophic lifestyle. Nevertheless, this strain represents the first chemolithomixotrophic bacterium within this genus. No fermentative growth was observed with any tested substrates.

Diazotrophic growth of strain MH2-6 was observed by the assimilation of ^15^N_2_ into cellular components (Figure S4), whereas the reference strain *Sulfurovum indicum* ST-419^T^ isolated from hydrothermal plumes [26], lacking the nitrogen fixation gene clusters, could not fix nitrogen. Furthermore, ^15^N_2_ fixation was fully inhibited upon ammonium addition, irrespective of the concentration (1 mM or 40 mM NH_4_Cl) (Figure S4). This finding was consistent with that of a previous study demonstrating nitrogen fixation is often repressed in the presence of exogenous nitrogen sources [30,38]. While in *Sulfurovum*, only *S. zhangzhouensis* zt1-1 from the pond sediments and *S. mangrovi* ST1-3^T^ from mangrove sediments have been reported to harbor the complete nitrogen fixation genes [16], suggesting their potential nitrogen fixation ability, but this has not been verified so far.

### 3.3. Genome Characteristics

The complete genome of strain MH2-6 was assembled into a circular chromosomal contig of 2,554,706 bases, and no plasmid was formed (Figure S5), which is different from the finding of the closest relative strain *S. mangrovi* ST1-3^T^ [39]. The DNA G+C content of strain MH2-6 was 43.5 mol%, which is similar to those of the genus Sulfurovum (Table 1). Gene annotation derived from RAST predicted 2598 genes, including 2514 protein encoding genes, 56 tRNA genes, and 15 rRNA genes. The pairwise ANI and dDDH values between strain MH2-6 and *S. mangrovi* ST1-3^T^ was 99.92% and 98.30%, respectively, indicating that the genome of strain MH2-6 was most similar to that of strain *S. mangrovi* ST1-3^T^, further supporting the 16s rRNA gene result. Comparative genome analysis between both strains based on the BPGA pipeline indicated that 2418 core genes were shared by both genomes, indicating that both strains shared a high percentage of common functional proteins.

#### 3.3.1. Carbon Metabolism

**Figure 4:**
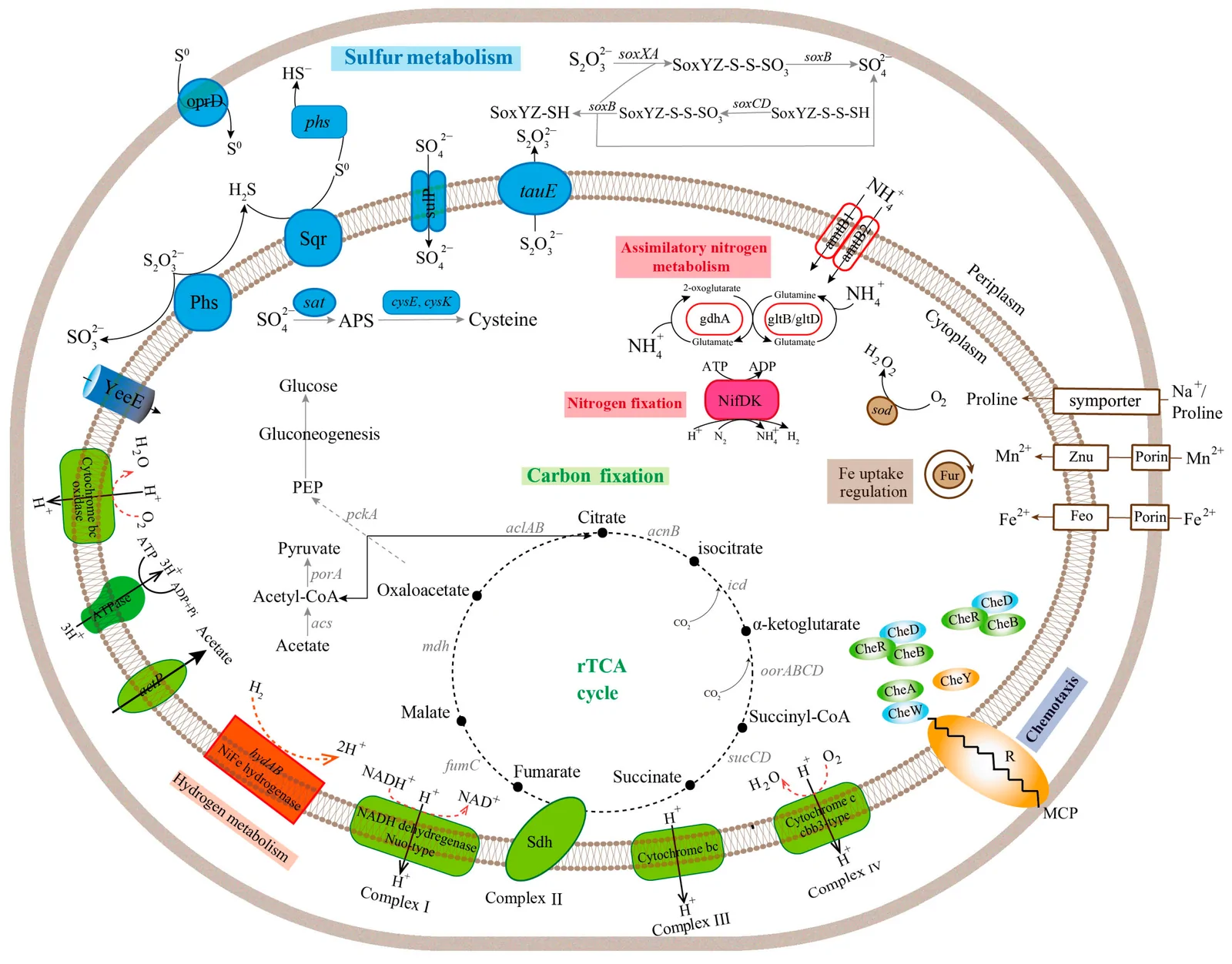
Metabolic reconstruction of strain MH2-6. The pathway was inferred form the genome by RAST and KEGG annotation. The model showcases the presence of genes involved in carbon, sulfur, nitrogen, hydrogen, oxygen, iron regulation pathways, and chemotaxis. Abbreviations of genes are stated in the main text.

The genome of strain MH2-6 contained all genes for the reductive citric acid cycle (rTCA) for carbon fixation, including pyruvate:ferredoxin oxidoreductase (PorABCD, EC 1.2.7.1), 2-oxoglutarate:ferredoxin oxidoreductase (OorABCD, EC 1.2.7.3), and ATP-dependent citrate lyase (AclAB, EC 2.3.3.8) (Figure 4). We also found a two subunit form of 2-oxoglutarate:ferredoxin oxidoreductase (OorAB, EC 1.2.7.3) in the genome. In Hydrogenobacter thermophilus belonging to the Aquificales, the five-subunit O_2_-tolerant forms of Oor and Por are employed under aerobic conditions, whereas the two-subunit O_2_-sensitive counterparts function during anaerobic growth [40]. Thus, the diverse enzymes may confer a survival advantage to the strain under dynamic hydrothermal environments. In addition, the glycolysis pathway was incomplete, and the detected genes were instead involved in the gluconeogenesis, supported by the presence of reverse-reaction enzymes including fructose-1,6-bisphosphatase (EC 3.1.3.11) and phosphoenolpyruvate carboxykinase (EC 4.1.1.49) (Table S1). The genome contained all genes of the Entner–Doudoroff pathway, whereas the pentose phosphate pathway was incomplete, lacking its oxidative phase.

#### 3.3.2. Sulfur Metabolism

The strain genome encoded the Sox multienzyme system for the oxidation of reduced sulfur compounds in the periplasm, which was organized into two clusters, SoxABXYZ and SoxCDYZ (Figure 4). Moreover, the genome harbored diverse types of sulfide: quinone oxidoreductase (Sqr), including type II, III, IV, VI, and flavocytochrome c sulfide dehydrogenase (FccB), both of which are responsible for sulfide oxidation [41]. Strain MH2-6 also harbored genes encoding sulfite dehydrogenase (SorAB), which catalyzes the oxidation of sulfite to sulfate [42]. No genes involved in dissimilatory sulfate reduction, such as adenylylsulfate reductase (AprAB) and sulfite reductase (DsrAB), were found in the genome. Sulfur compounds may also serve as substrates for assimilatory sulfate reduction for the synthesis of sulfur-containing amino acids such as cysteine, as supported by the detection of sulfate adenylyltransferase (Sat, EC 2.7.7.4), serine acetyltransferase (CysE, EC 2.3.1.30), and cysteine synthase (CysK, EC 2.5.1.47) (Table S1). A sulfate permease transporter (SulP) was present in the genome for importing sulfate into the cytoplasm, where it is assimilated [43,44]. In addition, we also identified several molybdopterin-dependent oxidoreductases that may be involved in the reduction or disproportionation of sulfur compounds, including thiosulfate reductase (Phs), polysulfide reductase (Psr), and sulfite reductase (Fsr) (Table S1).

#### 3.3.3. Sulfur Transporters

The genome contained genes encoding a sulfite exporter (TauE), and several cytoplasmic membrane sulfur-transferases YeeE/YedE (Figure 4), which acquires a sulfur atom from periplasmic polysulfide and donates it to the cytoplasm [45]. In addition, the genome also encoded several rhodanese-like sulfurtransferases that could participate in thiosulfate or sulfur disproportionation (Table S1), although the actual physiological role remains unresolved. Genes encoding other sulfur transferases with two rhodanese domains (SseA) were also detected in the genome. The genome also contained the gene dsrE, which encodes a small transmembrane protein in sulfur-oxidizing bacteria [20], but lacked the typical tusA gene that encodes a small sulfur-transporting protein belonging to the sulfotransferase family [10]. Additionally, a total of four genes encoding the outer membrane OprD-like porins were found in the genome (Table S1). The OprD family has been reported to facilitate the uptake of multiple specific substrates [46], so they may be involved in the activation and transport of sulfur into cells.

#### 3.3.4. Hydrogen Metabolism

The oxidation of H_2_ can be utilized as an energy source by the genome encoding a group I [NiFe]-hydrogenase (EC 1.12.99.6) (Figure 4), which is in accordance with the phenotypic data (Figure 2 A,B). In this process, the energy from the proton motive force is generated across the periplasmic membrane from H_2_ oxidation to protons and electrons [47]. Genomic analysis revealed that strain MH2-6 contained two copies of group I hydrogenase, possibly enabling adaptation to varying H_2_ concentrations, as has been observed in other Campylobacteria strains [35]. In addition, the strain genome harbored group II hydrogenases (EC 1.12.99.6), which may function in hydrogen sensing or energy conversion at low hydrogen concentrations [48].

#### 3.3.5. Nitrogen Metabolism

The genome lacked the complete denitrification pathway, including nitrate reductase (NapAB, EC 1.18.6.1), nitric oxide reductase (NorBC, EC 1.18.6.1), nitrite reductase (NirS, EC 1.18.6.1), and nitric oxide reductase (NosZ, EC 1.18.6.1). Physiology characterization showed that strain MH2-6 can use nitrate as the electron acceptor (Figure S1), suggesting that this process may occur via an unknown pathway. In contrast, strain MH2-6 contained all genes for nitrogen fixation, including those encoding the molybdenum-iron nitrogenase (NifHDK, EC 1.18.6.1) and its associated regulatory and accessory proteins (Table S1), which is consistent with its capacity for diazotrophic growth (Figure S4). Additionally, the genome harbored the amtB gene encoding for the channel protein responsible for ammonia uptake, as well as gltB (EC 1.4.1.13), which is involved in nitrogen assimilation required for amino acid metabolism.

#### 3.3.6. Oxygen Metabolism

The capacity for utilizing oxygen as a terminal electron acceptor was supported by the presence of multiple oxygen reductases (Figure 4). The genome encoded a cbb_3_-type cytochrome c oxidase (CcoNOQP, EC 1.9.3.1), a high-affinity terminal oxidase that enables growth under microaerobic and anaerobic conditions [49]. This cytochrome c oxidase has also been shown to be involved in O_2_ detoxification by efficiently scavenging trace oxygen [43]. The genome also encoded a cytochrome bd ubiquinol oxidase (CydAB, EC 1.10.3.-) (Table S1), another terminal oxidase with a high affinity for oxygen that can function at low oxygen conditions [26]. This is crucial for the bacterium’s adaptation to the oxygen-limiting conditions typical of deep-sea hydrothermal vents. In fact, growth experiments showed that the isolate can grow at O_2_ concentrations up to 20% (Table 1). Similar observations have been detected in Campylobacterota isolates [50], which may be attributed to the antioxidation system, as evidenced by the presence of superoxide dismutase (EC 1.15.1.1), catalase (EC 1.11.1.6), hemerythrin, and superoxide reductase (EC 1.15.1.2) (Table S1).

### 3.4. Transcriptomic Analyses Under Different Growth Conditions

**Figure 5:**
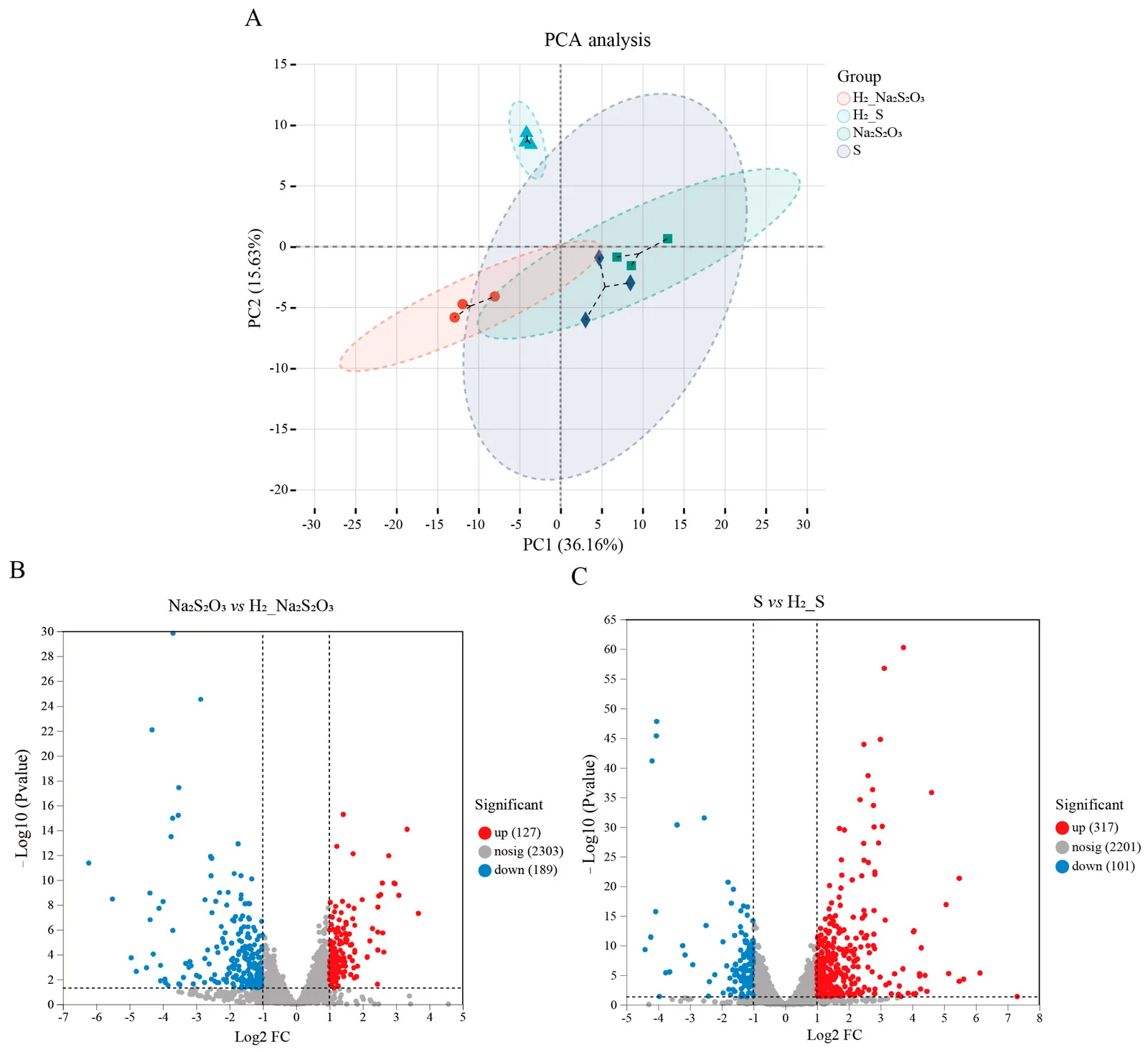
Transcriptomic profiles of strain MH2-6 under sulfur disproportionation and reduction. (**A**) A principal component analysis of all RNA-seq samples, showing how samples subjected to different treatments clustered separately. (**B**,**C**) Volcano plots display gene transcription patterns of strain MH2-6 under thiosulfate and elemental sulfur disproportionation compared to sulfur reduction, respectively. Significantly differentially expressed genes are highlighted in blue (upregulated) and red (downregulated).

To identify the key genes involved in sulfur disproportionation, we performed a transcriptomic analysis of strain MH2-6 harvested under four conditions, including thiosulfate or elemental sulfur reduction with hydrogen as the electron donor, as well as thiosulfate and elemental sulfur disproportionation. In total, 92, 184, 398 Illumina paired-end raw reads were generated (Table S2). After removing adaptor sequences, ambiguous nucleotides, and low-quality sequences, 90, 869, 000 clean reads remained. For all 12 samples, the percentages of sequence reads mapped to the strain genome ranged from 80.77% to 95.76%, with an average mapping rate exceeding 88% (Table S2). A principal component analysis (PCA) showed that the strong correlations of gene expression levels between biological replicates among treatments was convincing, with clear separation along the main axis (36.2% of the total variance) (Figure 5A). Furthermore, the expression patterns of four cultivated growth transcriptomes were assessed using a heat map, which showed a clear segregation across different experimental groups (Figure S6). Here we present the most important findings regarding sulfur metabolism. More detailed descriptions from the transcriptome analysis are provided in Tables S3 and S4.

**Figure 6:**
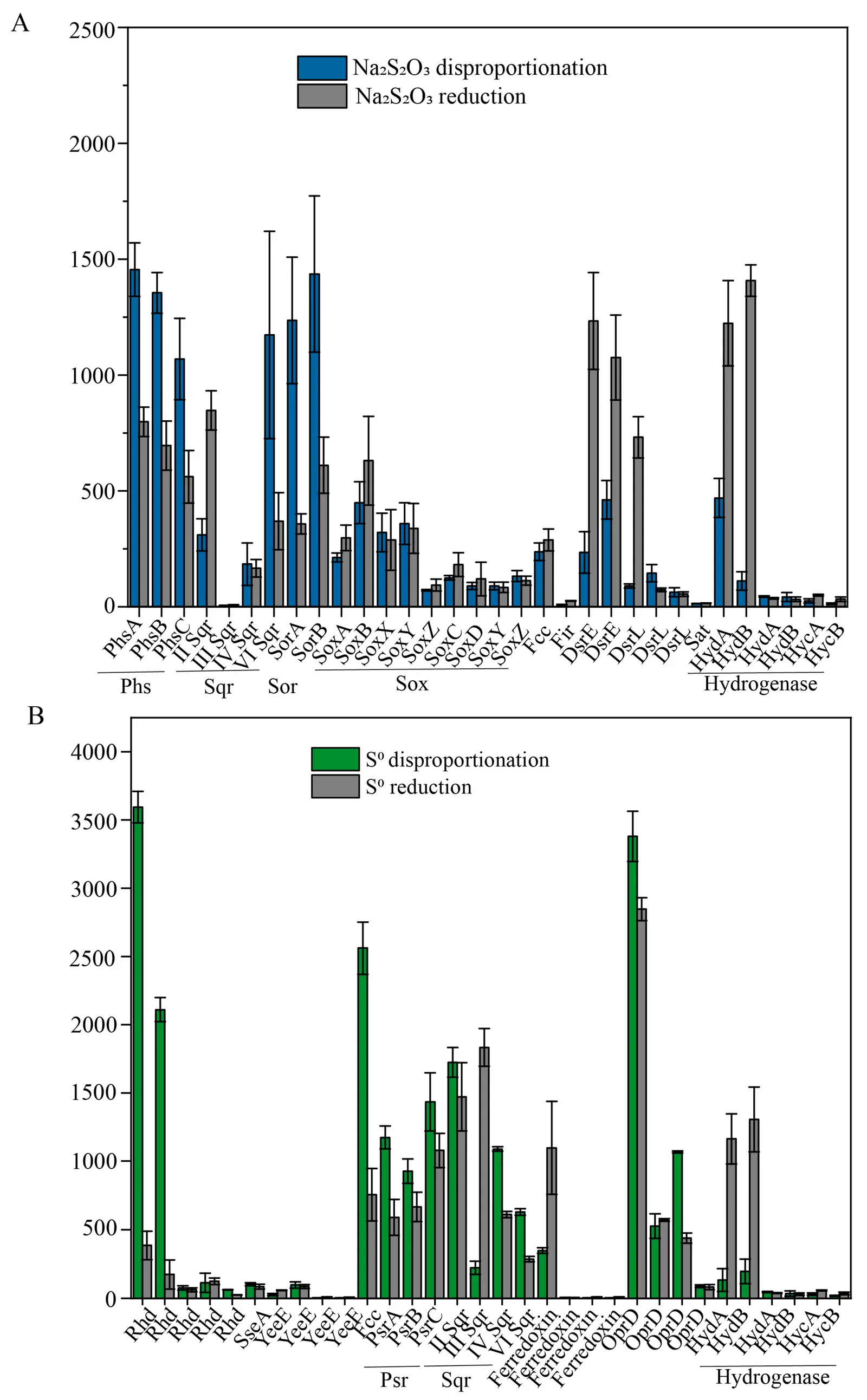
Relative transcript abundance of genes possibly involved in thiosulfate (**A**) and elemental sulfur (**B**) disproportionation of strain MH2-6 relative to the control conditions. Normalized gene expression is plotted as fragments per kilobase and million reads (FPKM). Error bars indicate the standard deviation from triplicates.

To identify the genes involved in thiosulfate disproportionation, a total of 127 genes were differentially up-regulated (expression level more than twice as high) and 189 genes were down-regulated (expression level less than twice as low) during the growth of strain MH2-6 by thiosulfate disproportionation compared to thiosulfate reduction (Figure 5B). The genes encoding thiosulfate reductases (PhsABC, MHY6962085-MHY6962087) were detected under both culture conditions, but showed significantly higher expression under sulfur disproportionation (Figure 6A), indicating that they may play a more important function for thiosulfate disproportionation. A recent study has reported that PhsAB in a deltaproteobacterial *Desulfurella amilsii* can cleave thiosulfate into sulfite and sulfide [51]. Furthermore, protein domain analysis showed that PhsA contained a two arginine protein translocation (TAT) signal peptide, thus standing for a dimeric periplasmic enzyme [35]. In addition, the gene encoding type VI Sqr (MHY6962014) was differentially highly expressed under thiosulfate disproportionation compared to thiosulfate reduction, indicating its possible involvement in thiosulfate disproportionation. Previous studies have reported that VI SQR enzyme can work in the sulfur reductive direction in *Sulfurovum* sp. NBC37-1 and *Thermovibrio ammonificans* [52,53]. Thus, as recently proposed for *Desulfurivibrio alkaliphilus* [51], it is possible that enzymes known to be involved in sulfur metabolism have an unexpected function. Moreover, genes encoding sulfite dehydrogenase (SorAB, MHY6960935, MHY6960936) were significantly highly expressed under thiosulfate disproportionation compared to sulfur reduction (Figure 6A), suggesting that part of the thiosulfate needs to be oxidized during the disproportionation process. However, some genes associated with typical sulfur oxidation, such as those encoding the Sox complex and Fcc exhibited very low expression levels under both conditions, suggesting that these genes may be less likely to participate in these processes. Compared to thiosulfate disproportionation, the gene encoding F420-dependent sulfite reductase (Fsr) that is likely related to sulfite reduction was expressed at a low level, but the genes encoding DsrE (MHY6961688, MHY6961686), which serve as a sulfurtransferase reported in sulfur-oxidizing bacteria [54], were highly abundant in cultures grown by thiosulfate reduction (Figure 6A), supporting their potential involvement in sulfur reduction. Given that DsrE harbored the conserved cysteine residues, this protein may function as a sulfur carrier protein in strain MH2-6. Another typical sulfur oxidation-associated protein, DsrL (MHY6961826), was also detected in high abundance under thiosulfate reduction conditions (Figure 6A), indicating that it may function in a reductive mode, as reported in the previous study [37]. Sat, which is proposed to be linked to the oxidative branch of sulfur disproportionation [10], was relatively lowly expressed under both conditions. Additionally, the transcript abundances of group I hydrogenase were relatively high (with a FPKM up to 1490) than those of group II hydrogenase (only 25 FPKM value) under thiosulfate reduction compared to sulfur disproportionation. These results indicate the group I hydrogenase can be essential to the hydrogen oxidation process, which is consistent with the physiological study.

For elemental sulfur disproportionation, a total of 317 genes were differentially up-regulated, and 101 genes were down-regulated compared to elemental sulfur reduction (Figure 5B). To date, the molecular markers involved in the elemental sulfur disproportionation pathway remain putative, although several enzymes of the dissimilatory sulfate reduction pathway, YTD gene cluster (*yedE*, *dsrE*, *tusA* and two hypothetical genes), molybdopterin oxidoreductases, rhodanese-like sulfurtransferase and shorter *aprB* gene (with a truncated tail) have been proposed as potentially involved [11,27]. Here, two rhodanese-like sulfurtransferases (MHY6960676, MHY6961006) were detected with differentially high abundances under elemental sulfur disproportionation compared to reduction (Figure 6B), which is consistent with the proteomic profiles of *Desulfurella amilsii* cultivated under S^0^-disproportionating conditions [51]. Other sulfur transferases associated with activation of insoluble sulfur compounds and their transfer into the cells such as SseA and YeeE exhibited relatively low expression levels under both conditions. Moreover, the gene encoding sulfide dehydrogenase (Sudh/Fcc, MHY6961351) showed significantly higher expression under elemental sulfur disproportionation than under reduction (Figure 6B), suggesting its potential involvement in the sulfur disproportionation process. A previous study has indicated that a homologue of the gene encoding sulfide dehydrogenase, which also catalyzed polysulfide reduction to sulfide, can increase the availability of sulfide from protons and sulfur [55]. However, its role in sulfur disproportionation requires further validation. In addition, polysulfide reductases (PsrABC, MHY6961610–MHY6961612) were highly expressed under both conditions, but showed significantly higher expression under elemental sulfur disproportionation (Figure 6B), indicating they may play a more important role in sulfur disproportionation. Compared to elemental sulfur disproportionation, the gene encoding NADH-dependent sulfur reductase (NSR, MHY6961369), belonging to the pyridine nucleotide disulfide oxidoreductase family, was detected in high abundance under sulfur reduction conditions (Figure 6B), which is consistent with the previous study [52]. The NSR enzyme uses the FAD coenzyme as an electron shuttle to mediate electron transfer from NAD(P)H to a cysteine (Cys) residue in a redox-active disulfide bridge [35]. In addition, ferredoxin (MHY6959966) also showed high abundance under elemental sulfur reduction condition, further supporting that this enzyme functions as an NADH-dependent ferredoxin: NADP^+^ oxidoreductase, as reported in other anaerobes [41]. Moreover, the OprD porins were detected with high abundances under both conditions (Figure 6B), which has been shown to be involved in the utilization of solid S^0^ in *Sulfurimonas denitrificans* DSM 1251 and *Sulfurimonas* sp. CVO by the transcriptomic and/or proteomic analyses [56,57]. However, this hypothesis would require further research to confirm the results. Additionally, the transcript abundances of group I hydrogenase were relatively higher under elemental sulfur reduction compared to disproportionation (Figure 6B), which is consistent with the above result with thiosulfate as electron acceptor.

## 4. Conclusions

This study provides the first physiological, genomic, and transcriptomic analysis of a chemolithomixotrophic microorganism grown under sulfur disproportionation and reduction conditions. Strain MH2-6 has well adapted itself to hydrothermal vent environments, mainly via hydrogen oxidation, sulfur oxidation, reduction, disproportionation, nitrogen fixation and carbon fixation, suited to environments that containing abundant hydrogen, diverse inorganic sulfur species, limited carbon dioxide, and fluctuating oxygen and pH conditions. A schematic model of sulfur metabolism in *Sulfurovum* strain MH2-6 was generated based on genomic analysis, providing the first detailed description of the genus *Sulfurovum*. Transcriptomic analysis revealed that proteins related to thiosulfate reductase as well as quinone oxidoreductase and sulfite dehydrogenase, well known for their involvement in the oxidation of reduced sulfur compounds, were more abundant in cultures grown via thiosulfate disproportionation. In addition, several proteins possibly involved in elemental sulfur disproportionation, including those encoded by the rhodanese-like sulfurtransferase, were also found. Further biochemical and enzymological characterization will be needed to elucidate the functions of the putative enzymes identified in this study. Regardless, these features may enable these organisms to be widespread and highly adaptable to different deep-sea hydrothermal vent fields.

## Figures and Tables

**Table 1:** Comparison of characteristics among *Sulfurovum* sp. MH2-6 and related type strains within the genus *Sulfurovum*.

Characteristic	1	2	3	4	5
Origin	Hydrothermal Sediment, South Mid-Atlantic Ridge	Mangrove Sediment, Jiulong River Estuary	Pond Sediment, Zhangjiang River Estuary	Mangrove Sediment, Xixi River Estuary	Hydrothermal Chimney, Central Indian Ridge
Shape	Rod	Rod	Rod	Rod	Rod
Doubling time under optimal (h)	2.5	4.6	ND	ND	3.2
Temperature range (Optimal T) (°C)	4–50 (32)	4–45 (35)	10–45 (32)	15–45 (32)	15–37 (33)
pH range (Optimal pH)	5.0–8.0 (6.0)	5.0–8.5 (7.0)	4.5–8.5 (6.0–6.5)	5.0–8.5 (6.0–6.5)	5.5–8.6 (6.0)
NaCl range (Optimal) (% *w*/*v*)	1.0–5.0 (3.0)	0–8.0 (4.0)	0–7.0 (1.5)	0.5–5.0 (2.5)	2.0–4.0 (2.5)
Maximum O_2_ concentration (%)	20	20	20	15	3
Electron donor	H_2_, S_2_O_3_^2−^, HS^−^, SO_3_^2−^	H_2_	H_2_, S_2_O_3_^2−^	H_2_, S_2_O_3_^2−^	H_2_
Electron acceptor	O_2_, S^0^, S_2_O_3_^2−^, NO_3_^−^, SO_4_^2−^	O_2_, S_2_O_3_^2−^, S^0^	O_2_, S_2_O_3_^2−^, S^0^	O_2_, S_2_O_3_^2−^, S^0^	S^0^, NO_3_^−^, S_2_O_3_^2−^
Sulfur disproportionation	+	+	ND	ND	ND
Utilization of organic compound as carbon sources	+	−	−	−	−
G+C content (mol%)	43.5	43.6	39.4	39.1	42.6

Strains: 1, *Sulfurovum* sp. MH2-6; 2, *S. mangrovi* ST1-3^T^; 3, *S. zhangzhouensis* zt1-1^T^; 4, *S. xiamenensis* XTW-4^T^; 5, *S. aggregans* Monchim33^T^. Strain 1 (MH2-6) and strain 2 (*S. mangrovi* ST1-3^T^) represent the same species. +, Positive; −, Negative, ND, Not determined.

## Data Availability

The 16S rRNA gene sequence and complete genome sequence of strain MH2-6 have been deposited in GenBank under accession numbers OQ102629.1 and CM178170.1, respectively. The transcriptome data has been deposited in the NCBI Sequence Read Archive (SRA) under the accession numbers SRR39141921 to SRR39141932.

## Supplementary Materials

The following supporting information can be downloaded at: https://www.mdpi.com/article/10.3390/microorganisms14091916/s1. Figure S1: The growth characterization of *Sulfurovum* sp. MH2-6 with hydrogen as the electron donor and nitrate as the sole electron acceptor. Error bars indicate the standard deviation from triplicates; Figure S2: The growth characterization of strain MH2-6 with hydrogen as the electron donor and sulfate as the sole electron acceptor. Error bars indicate the standard deviation from triplicates; Figure S3: Effects of different carbon sources on cell density. Cells were cultured with yeast extract, tryptone, succinate, glucose, or fructose as the sole carbon source. Error bars indicate the standard deviation from triplicates; Figure S4: Determination of nitrogen fixation activity of strain MH2-6 based on the ^15^N incorporation assay, with H_2_ as the sole electron donor and O_2_ as the electron acceptor. *Sulfurovum indicum* ST-419^T^, which lacked the nif gene cluster served as a control; Figure S5: Circular genome map of *Sulfurovum* sp. MH2-6. The outermost ring indicates genome size. The second and third rings show protein-encoding genes on the positive and negative strands, respectively. The fourth ring denotes rRNA and tRNA. The fifth ring represents G+C content; and the innermost ring shows the GC-skew value; Figure S6: A expression patterns was evaluated using an expression heatmap, with the gene cluster analysis done with log10 (FPKM + 1) values; Table S1: The metabolic pathways of strain MH2-6 were analyzed based on RAST annotation; Table S2: Summary of Illumina RNA-seq data in this study. Table S3: A total of 316 differentially expressed genes, including 127 upregulated and 189 downregulated, under thiosulfate disproportionation and reduction conditions. Table S4: A total of 418 differentially expressed genes, including 317 upregulated and 101 downregulated, under elemental sulfur disproportionation and reduction conditions.
