# Supplementary material for: Insights into the Versatile Sulfur Metabolism of Sulfurovum sp. MH2-6 Isolated from Deep-Sea Hydrothermal Vent Environments
Source: Microorganisms. 2026 Aug 30;14(9):1916. doi: 10.3390/microorganisms14091916 (PMC13609358; doi:10.3390/microorganisms14091916)
Supplement: Supplementary file 1 [file microorganisms-14-01916-s001.zip › Supplemental data.pdf]

Insights into the Versatile Sulfur Metabolism of *Sulfurovum* sp. MH2-6 Isolated from Deep-Sea Hydrothermal Vent Environments

Liang Cui [1,2,†], Shasha Wang [1,2,*,†], Xuewen Gao [1,2], Rongfeng Hong [1,2], Zongze Shao [1,2,3] and Lijing Jiang [1,2,*]

[1] Key Laboratory of Marine Genetic Resources, Third Institute of Oceanography, Ministry of Natural Resources of China, Xiamen 361005, China; cuiliang@tio.org.cn (L.C.); gaogaoxw@stu.xmu.edu.cn (X.G.); 13808528741@163.com (R.H.); shaozz@163.com (Z.S.)
[2] State Key Laboratory Breeding Base of Marine Genetic Resources, Third Institute of Oceanography, Ministry of Natural Resources of China, Xiamen 361005, China
[3] Fujian Ocean Innovation Center, Xiamen 361102, China
* Correspondence: wangshasha@tio.org.cn (S.W.); jianglijing@tio.org.cn (L.J.)
† These authors contributed equally to this work.

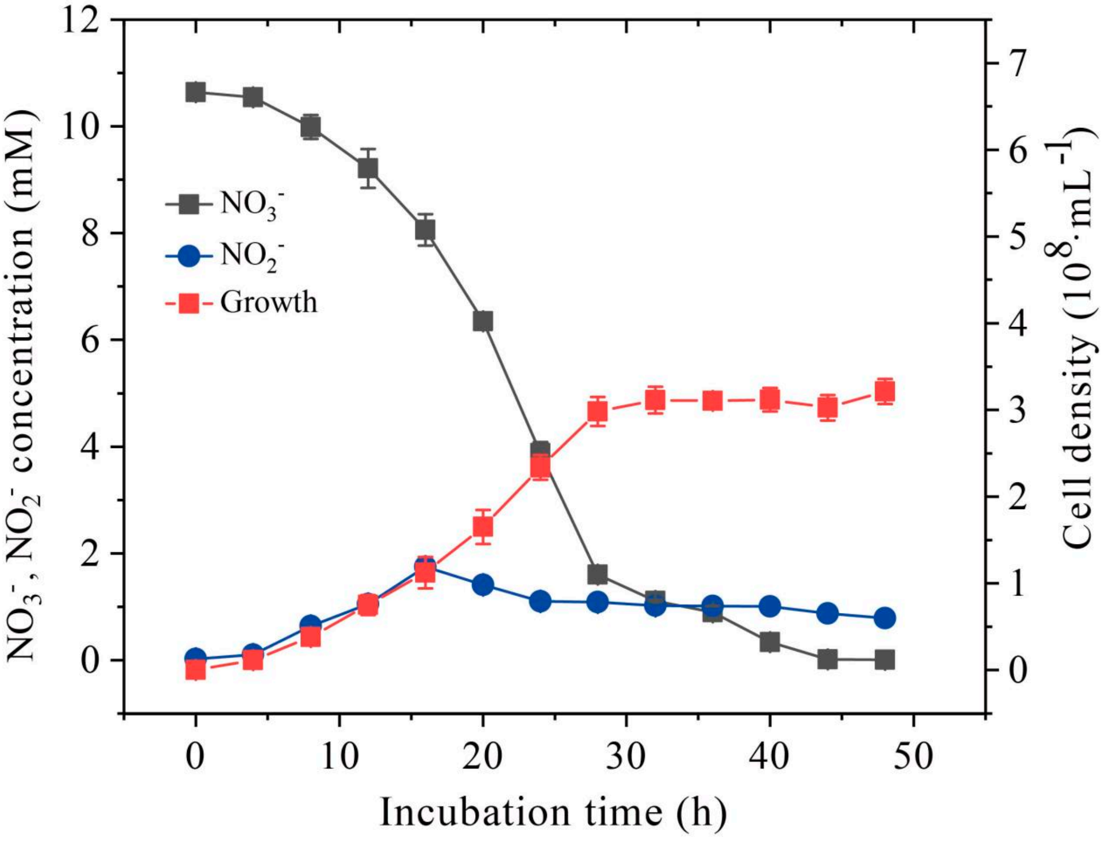

Figure S1. The growth characterization of *Sulfurovum* sp. MH2-6 with hydrogen as the electron donor and nitrate as the sole electron acceptor. Error bars indicate the standard deviation from triplicates.

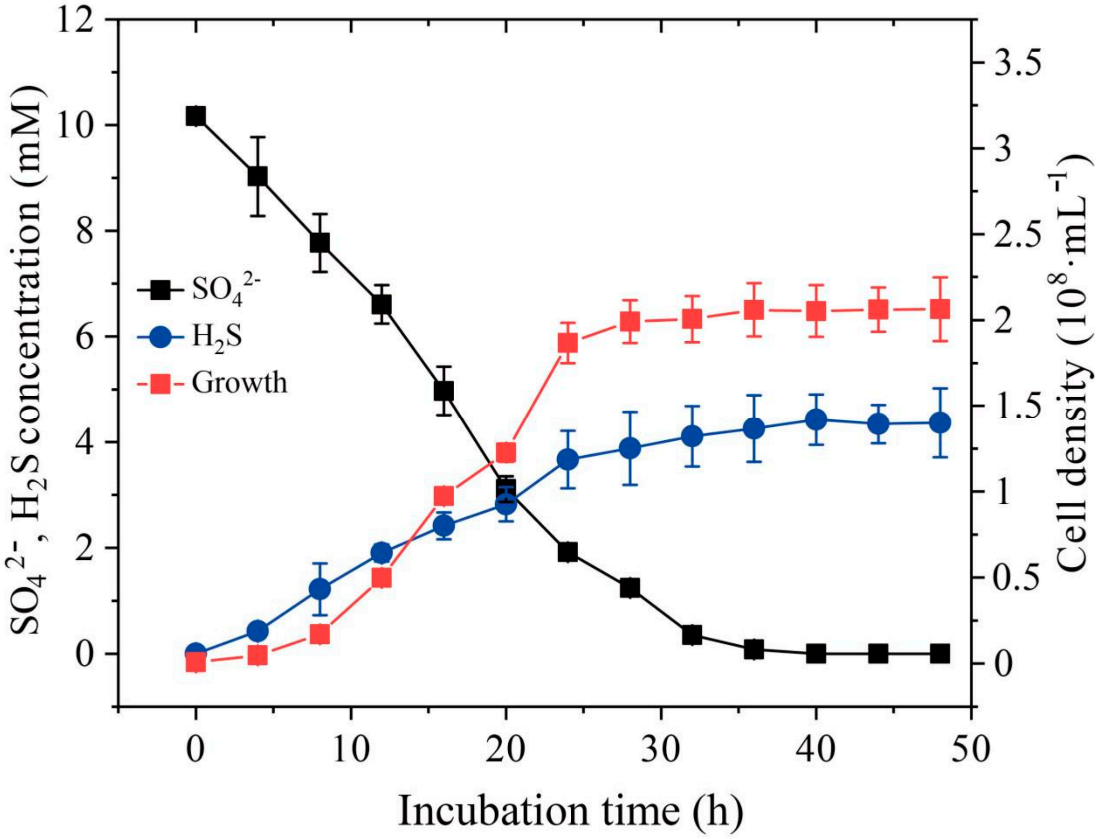

Figure S2. The growth characterization of strain MH2-6 with hydrogen as the electron donor and sulfate as the sole electron acceptor. Error bars indicate the standard deviation from triplicates.

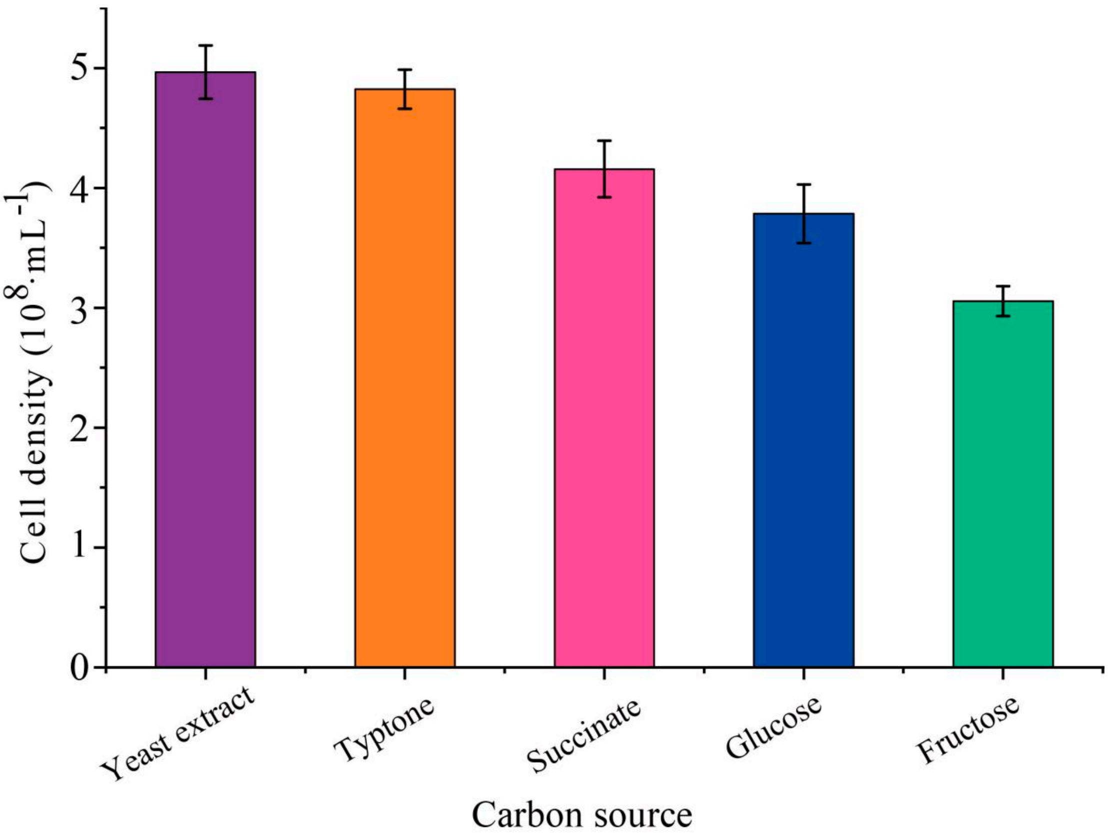

Figure S3. Effects of different carbon sources on cell density. Cells were cultured with yeast extract, tryptone, succinate, glucose, or fructose as the sole carbon source. Error bars indicate the standard deviation from triplicates.

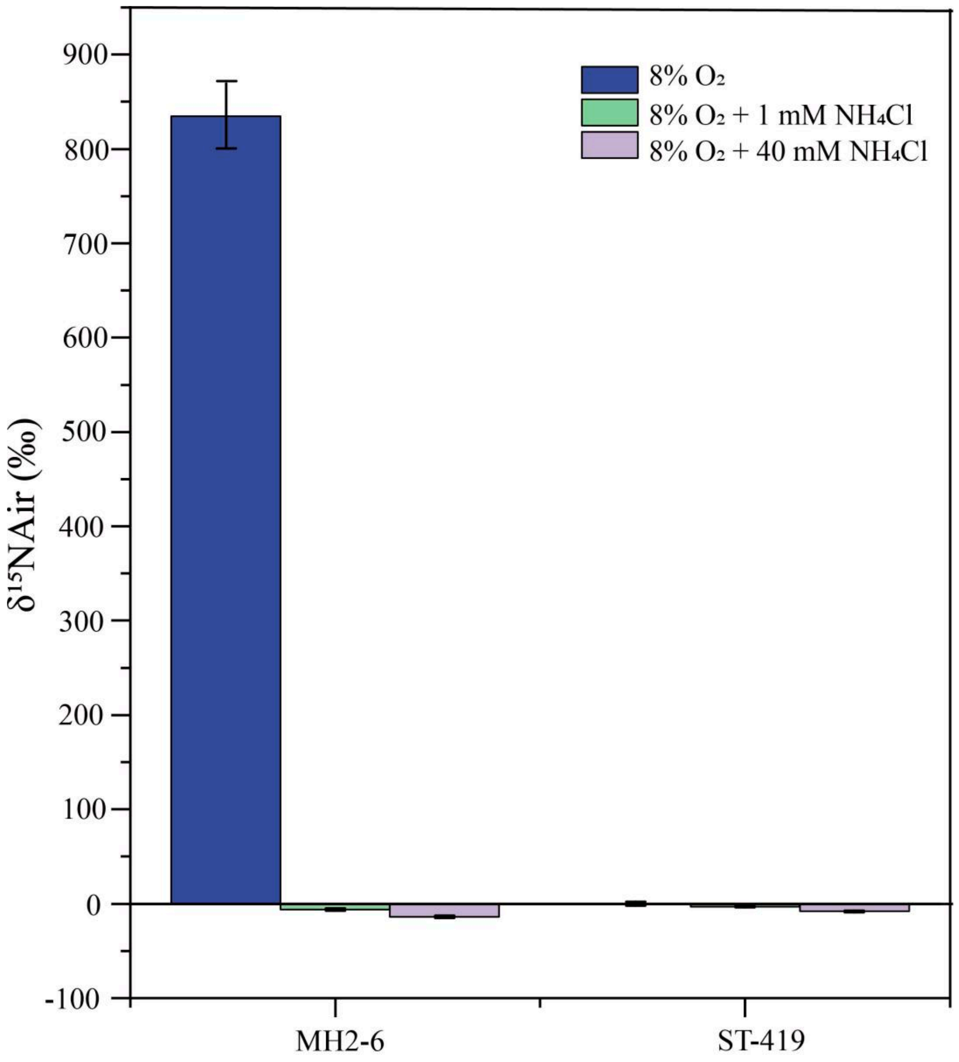

Figure S4. Determination of nitrogen fixation activity of strain MH2-6 based on the ^{15}N incorporation assay, with H_2 as the sole electron donor and O_2 as the electron acceptor. *Sulfurovum indium* ST-419^T which lacked *nif* gene cluster served as a control.

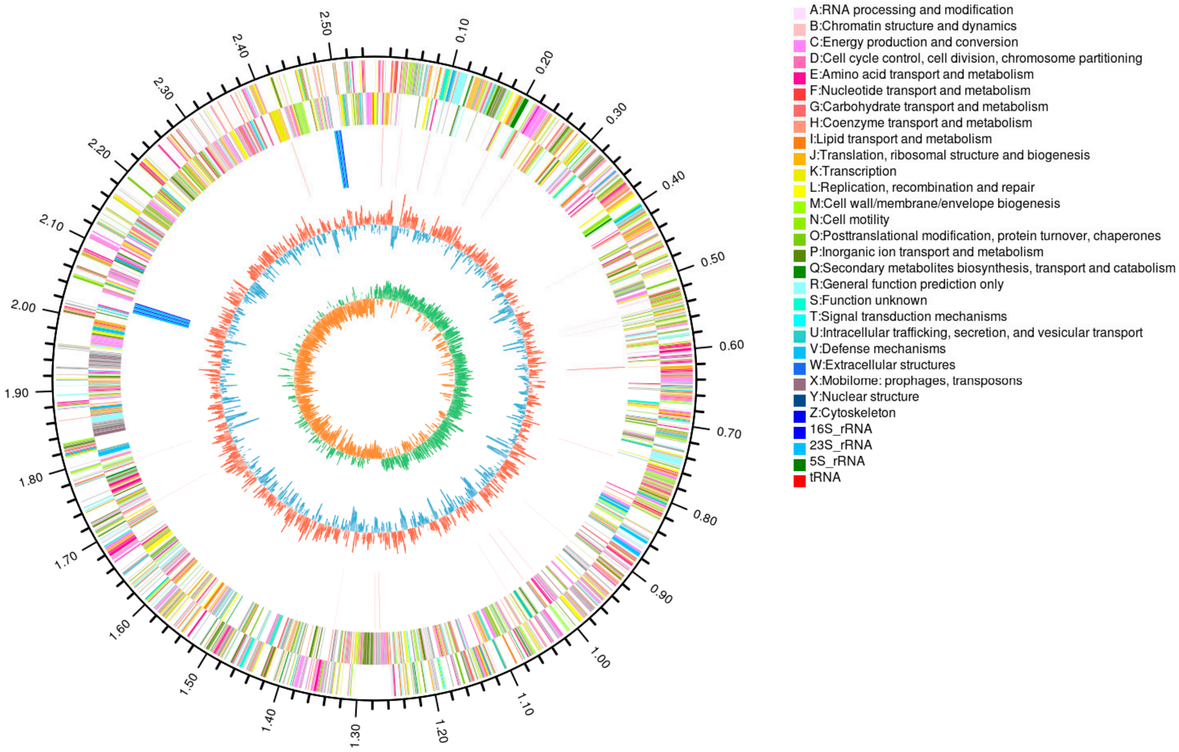

Figure S5. Circular genome map of *Sulfurovum* sp. MH2-6. The outermost ring indicates genome size. The second and third rings show protein-encoding genes on the positive and negative strands, respectively. The fourth ring denotes rRNA and tRNA. The fifth ring represents G+C content; and the innermost ring shows the GC-skew value.

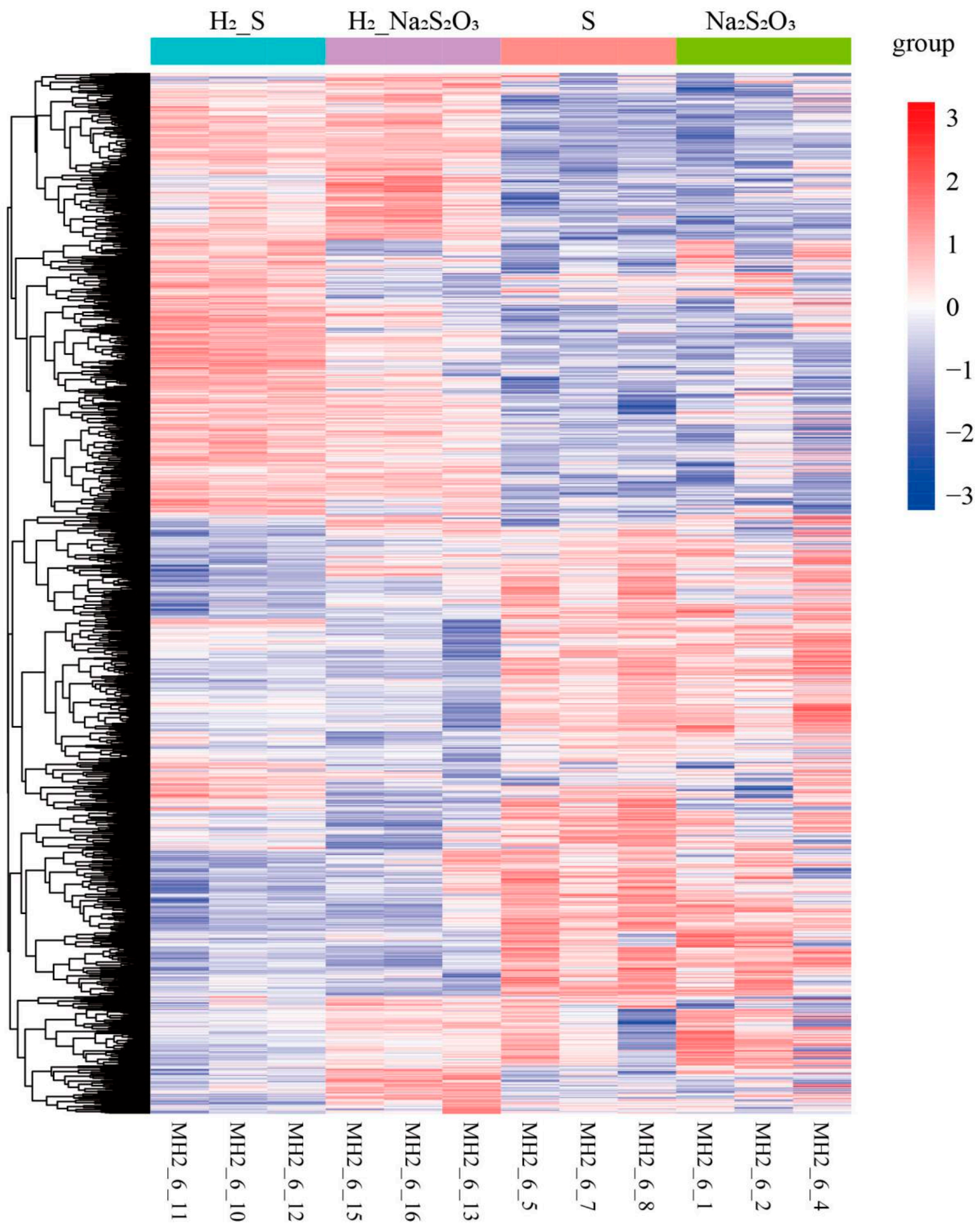

Figure S6. A expression patterns was evaluated using an expression heatmap, with the gene cluster analysis done with log10 (FPKM+1) values.
